# Tailoring a Digital Mental Health Program for Patients With Sickle Cell Disease: Qualitative Study

**DOI:** 10.2196/44216

**Published:** 2023-04-06

**Authors:** Cara Nikolajski, Julia O'Brien, Emily Nardo, Eva Szigethy, Charles Jonassaint

**Affiliations:** 1 Center for High-Value Health Care University of Pittsburgh Medical Center Pittsburgh, PA United States; 2 Department of Health and Community Systems School of Nursing University of Pittsburgh Pittsburgh, PA United States; 3 Center for Research on Health Care University of Pittsburgh Pittsburgh, PA United States; 4 Center for Behavioral Health and Smart Technology University of Pittsburgh Pittsburgh, PA United States; 5 Department of Psychiatry School of Medicine University of Pittsburgh Pittsburgh, PA United States

**Keywords:** mental health, sickle cell disease, digital health, cognitive behavioral therapy, digital cognitive behavioral therapy

## Abstract

**Background:**

Depression and other mental health disorders are prevalent among people living with chronic health conditions. Although digital cognitive behavioral therapy (CBT) is considered an effective treatment, African American individuals are less likely to engage in and adhere to digital therapies for mental health disorders compared with White individuals.

**Objective:**

The aim of this study was to understand digital CBT mental health treatment perceptions and preferences of African American individuals with sickle cell disease (SCD).

**Methods:**

African American individuals with SCD from various US locations were invited to participate in a series of focus groups. Participants were introduced to a health coach–supported mental health app and then asked a series of questions about the usability and appeal of the program as well as, more generally, what would make a digital mental health program effective for them. The authors reviewed the focus group transcripts and conducted a qualitative analysis of the results.

**Results:**

A total of 25 people participated in 5 focus groups. Overall, 5 primary themes emerged regarding how app content and related coaching could be modified to enhance digital CBT engagement. These themes included connection with others living with SCD, the personalization of app content and coaching, characteristics of coaches, journaling and pain tracking, and considerations for optimal engagement.

**Conclusions:**

Enhancing the user experience by making digital CBT tools relevant to patient populations is critical for optimizing program engagement and its uptake. Our findings highlight potential strategies to modify and design digital CBT tools for users with SCD and may also be applicable to patients with other chronic conditions.

**Trial Registration:**

ClinicalTrials.gov NCT04587661; https://clinicaltrials.gov/ct2/show/NCT04587661

## Introduction

### Background

African American individuals are more likely to experience severe depression than White individuals but less likely to receive treatment [1,2]. Depression and other mental health disorders are even more common in African American individuals living with chronic health conditions [3,4]. One particularly vulnerable population is individuals with sickle cell disease (SCD), a heritable condition that affects millions of people worldwide and >100,000 people in the United States, most of whom are of African descent [5]. Among patients with SCD, 38.8% present with mental health disorders [6]. A review of the literature on mental health symptoms in SCD found that 22% to 57% of individuals with SCD report clinically significant depressive symptoms depression when screened [7]. Similarly, anxiety disorders are also high in this population. In a worldwide study of quality of life among patients with SCD, 38% reported having symptoms of anxiety [8].

Depression and anxiety disorders not only lead to poorer quality of life but in patients with SCD, depression is associated with increased pain frequency and duration, pain-related hospitalizations, opioid dependence, medical costs, and risk of mortality [9,10]. Despite the high prevalence of mental health disorders and the links among depression, anxiety, and SCD-related health outcomes, mental health is rarely assessed, let alone treated as a part of routine SCD care. This is a major gap in care, given that effective and cost-efficient mental health treatments, such as cognitive behavioral therapy (CBT), exist but are often not part of comprehensive SCD care [11-15].

There are several barriers to the implementation of face-to-face behavioral health care in SCD clinics. Access to CBT is limited because of the expense of providing face-to-face therapy in low-resource clinical settings and a shortage of available CBT-trained therapists, especially those who are culturally competent to work with hard-to-reach minority populations. Even when CBT is available, therapy is time-consuming and requires traveling to a clinic, which is challenging for adult patients with major social and economic challenges [16-18] and for the parents of adolescent patients [19].

However, technology may be able to overcome these barriers and increase access to CBT among patients with SCD. Over the past 2 decades, face-to-face CBT has been adapted so that it can be delivered over the phone; over the internet via a desktop computer; and more recently, via mobile devices. These programs often combine a digital education component with personalized support, usually through a health coach [20,21]. Digital CBT makes it possible to deliver high-quality, evidence-based behavioral mental health treatments at scale in SCD care, in which face-to-face CBT may not be feasible [14,22]. The main limitation of digital CBT in SCD care is that its uptake and engagement are often poor among minority race and ethnic groups, and even when made available, patients with SCD may not use digital CBT [13]. One digital CBT trial showed that African American individuals in primary care with depression and anxiety were less likely than White individuals to start the digital CBT program (75% vs 87%) and complete all available modules (29% vs 43%) [12,13]. One potential explanation for racial differences in digital CBT uptake and engagement is that Black patients may find that the content is not relevant or relatable to them and does not represent their real-world cultural and health experience [14]. However, no qualitative studies have examined barriers to and strategies for improving the implementation of digital CBT in routine SCD care.

### Objectives

The objective of this study was to conduct a series of qualitative focus groups to determine the factors that promote the successful implementation and uptake of a health coach–supported digital CBT program for mental health in SCD care. Among patients with SCD, adolescents and young adults are at high risk for depression and anxiety [23-25], have an increased risk of complications [26], and have the highest rate of acute care encounters and rehospitalizations [27]. Therefore, we focused on improving digital CBT uptake and engagement primarily among adolescents and young adults with SCD. The focus group findings described herein will be used to tailor an existing health coach–supported digital CBT program to enhance its implementation among individuals with SCD.

## Methods

### Study Design

We used a convenience sampling strategy, working with SCD-focused community-based organizations to recruit focus group participants residing in diverse locations across the United States. Participants were referred to the study team through these organizations and were contacted by the study team via email. Owing to the geographical dispersion of our sample and to maintain the safety of participant and study team during the COVID-19 pandemic, focus groups were internet based. The target participants for these focus groups were adolescents or young adults who reported having an “interest in or experience with mental health.” The target size for these focus group ranged from 8 to 10 participants. The focus group facilitator verbally provided potential participants with information regarding elements of informed consent before the start of each focus group (eg, risks and benefits, participation procedures, data use, and the voluntary nature of the project), and after verbal consent was obtained, the focus groups proceeded using an institutional review board–approved focus group guide. Our focus group guide included questions regarding the impact of SCD on mental health and their attitudes and preferences regarding digital tools designed to support management of mental health symptoms. During the focus groups, participants were shown screenshots of a health coach–supported digital CBT program called RxWell, which is offered by the University of Pittsburgh Medical Center Health Plan, to promote conversation related to the program’s elements, layout, and graphics. The app is publicly available on the Google Play and Apple app stores and is free of cost for University of Pittsburgh Medical Center Health Plan members. For each element of the digital CBT program (ie, in-app techniques, symptom tracking, and digital coaching), participants were asked to express their general perceptions of each element, why they would or would not engage with various program components, their perceptions of the look and relatability of the program, and their preferences regarding coach communication. Focus group discussions were transcribed verbatim and participants received a US $30 food service gift card for their participation.

### Qualitative Analysis

A total of 3 coders participated in the analysis. The primary qualitative coder led the development of the initial code book. The codebook draft was tested by 2 secondary coders who used the first 2 transcripts. All 3 coders then met to talk through and to finalize the codebook. The primary and one of the secondary coders coded all 5 transcripts, meeting after each focus group to discuss similarities and differences. The third coder adjudicated all the disagreements. We did not record precise frequencies of comments because of the difficulty in quantifying focus group dynamics (eg, a participant may be the first to comment, with others providing affirmation through head nods or other forms of nonverbal agreement). Thus, we have chosen to use words, such as “some” or “many,” to provide a meaningful reflection of the commonality of responses without quantification. Individual comments are noted as such or provided as a quotation.

### Ethics Approval

The study protocol was approved by the University of Pittsburgh Human Research Protections Office (Study20070307) and was conducted in compliance with the ethical standards of the responsible institution on human participants as well as in accordance with the Declaration of Helsinki.

## Results

### Participant Characteristics

We recruited 25 African American individuals with SCD for this study. On the basis of their availability, the individuals participated in one of five 90-minute focus groups conducted between December 2020 and March 2021. The focus group size ranged from 3 to 9 participants. Participants were, on average, aged 23 years (range 16-47 years with 1 missing); 52% (13/25) were female, 32% (8/25) were male, 4% (1/25) were nonbinary, and 12% (3/25) did not disclose their gender. Only one participant had a previous exposure to RxWell.

### Salient Themes

#### Overview

In total, 5 primary themes emerged, suggesting that app content and coaching could be modified to enhance digital CBT engagement in this population. Each theme is described in the subsequent section along with examples of quotes from focus group participants that align with each theme (Textbox 1).

**Focus group themes and example quotes.**

**Connection with others living with sickle cell disease**

- “I’ve always struggled with finding like-minded people instead of trained professionals...I think it would also be beneficial if you had groups on there [the app] with people who have sickle cell that can share their thoughts and experiences.”
- “I think it would also be cool if there was, like, a blog portion where...people with sickle cell specifically, could jot their thoughts...‘This is how I deal with this,’ that type of thing.’”
- “If this app was generalized [meant for general audiences], maybe there can be a link to the specialized sickle cell app.”

**Personalization of app content and coaching**

- “This makes me think of data, and chatbots, and a bunch of things actually...The coach was recommending different, like, techniques that you could so...the app could also do that based on how your depression trends have been. Like, maybe it’ll encourage you more to pick up certain different techniques to do more often.”
- “I feel like a welcome screen...you could put in all of that information...how old are you, your name, if you have a chronic illness...”
- “Instead of having me go to the app, you know, maybe my coach can text me and say, boom, ‘Okay [participant’s name], how are you doing today? How are you feeling?’...Because some people feel like they don’t have anybody.”

**Characteristics of coaches**

- “For me to use it, it [the coach] would have to be...a [sic] actual trained professional not just, um, some customer service type. I want it to be someone who went to school for it, you know?”
- “I probably prefer someone who has experience with conditions that I have. So, if they can relate better – whether they have the condition themselves, or they’ve studied, or anything like that...For me personally, I think as long as they, like, have a good understanding of what I’m going through...should be good.”
- “I also really like the coaching aspect...I think, like, if they were to have, like, a personalized bio—what would be most appealing is, like, just stating why users should trust them and why we should take their advice.”
- “I already built my trust with my other coach, and I have to do it again with another coach. So, one set coach would be good like everybody else is saying.”

**Journaling and pain tracking**

- “I’ve taken a lot of surveys that say, like, ‘not at all’ and ‘several days’ like that. I think I would just personally change how that’s constructed a little bit to give it more of a journal feel.”
- “It could be useful to have a pain scale as well on the app. So, you can kind of track your pain and if it’s connected to your mood.”

**Considerations for optimal engagement**

- “I have a really short attention span for things, so I get really bored with things easily. So, um, I really don’t use those parts of the app [the techniques]. But another part of the app is you get connected with a coach...My coach just kind of checks in and asks me questions about my goals...That’s been really helpful to always have someone just checking in...I don’t want to say I’m advanced, but I’ve been kind of doing this CBT work and stuff with my therapists for years. So, none of this is new to me. And rehashing it is not very helpful to me.”
- “I also think with our age group, specifically, I think it could be really helpful because most people our age...do a have smartphone. They do use apps. And I think that a person might be more inclined to use this app before they would go to, like, a therapist or speak with someone in person.”
- “I don’t know if I would sustain the use though...I guess it depends on everybody’s need, right?”

#### Connection With Other People With SCD

Living with SCD was described as an isolating experience, and some participants expressed having difficulty finding opportunities to connect with individuals with similar lived experience. Some participants mentioned the potential benefit of a message board or chat function, in which individuals with SCD could connect and discuss experiences of dealing with the disease and depression, anxiety, or stress. Another group of participants acknowledged that it may be difficult to tailor the digital CBT program for people with a specific disease or condition. They suggested including links to alternative sites and resources within the app so that users could easily navigate as desired.

#### Personalization of App Content and of Coaching

All focus groups described elements of personalization that they felt would enhance their engagement with the digital CBT program. Most ideas pertained to the coaching component, but a few participants mentioned small ways in which the app itself could be personalized, such as including the use of the user’s name throughout.

There were a number of suggestions for coaching personalization: (1) adding an intake survey that allows users to provide personal information and preferences that the coaches can use to tailor the experience; (2) integrating a chatbot into the program that could collect user data and further personalize the experience; (3) integrating some discussion of SCD into coach communications but not focusing on SCD as the sole contributor to depression, anxiety, or stress; (4) having coaches send text messages occasionally to check in with users so that they do not always have to receive communications through the app; and (5) using other forms of messaging beyond typical coach check-in messages (eg, motivational quotes and pictures) to engage and support the mental health of users.

#### Characteristics of Coaches

The participants had mixed views on the coach’s characteristics and experience. Some people preferred someone with a formal mental health training, whereas others wanted to connect with someone with similar lived experience (eg, one with SCD or another chronic condition). Otherwise, participants had little preference related to coach’s characteristics, as long as the coaches were down-to-earth and nonjudgmental and did not use scripted responses. In addition, some participants felt that access to a brief coach biography would be beneficial for building a trusting relationship with the coach. Finally, the participants agreed that engaging with a single coach over time would be optimal.

#### Journaling and Pain Tracking

Several participants mentioned the potential benefits of incorporating a journaling element into the digital CBT program to provide more personal narrative regarding their day-to-day experiences and contributors to worsening or improved mood. This was also mentioned as an opportunity to log more detailed information beyond the standard validated depression and anxiety symptom assessments used in the digital CBT program. In addition, several participants acknowledged that pain was a common SCD symptom and contributed to the exacerbations of mental health symptoms; thus, in-app pain tracking and medication reminders would provide additional benefits.

#### Considerations for Optimal Engagement

Engagement with the digital mental health program involves interacting with the app to learn and practice new techniques, the completion of monthly symptom assessments, and regular engagement with a coach. Although more frequent engagements are recommended, there are no clear guidelines on the optimal level of engagement. There was substantial variability among the participants in terms of reporting how much they would engage with the program. Some participants mentioned that they might only engage with the program once or twice a week, only when needed, for a short period, or not at all, whereas others with more chronic depression or anxiety symptoms would consider using the app for a longer period. These differences in user needs may require a reconsideration of how to optimize engagement for various subgroups of digital CBT tool users.

## Discussion

### Principal Findings

People with SCD are at a high risk of developing mental health disorders [7,10,28,29]. Digital CBT has been found to be an effective, scalable, and cost-efficient method for treating mental health problems among patients with diverse psychiatric disorders [30,31]. Despite positive evidence for digital CBT, African American patients are less likely to use and engage with these types of interventions compared with White patients [13]. To fill this gap and to guide the design of these programs, it is critical to request insights from patients regarding what would be engaging and useful to them. Therefore, this study conducted a series of focus groups with adolescents and adults with SCD to better understand their perceptions of digital CBT, identify features that would enhance engagement in our digital CBT program, and take initial steps in developing a framework to guide the design of digital interventions that target underrepresented groups, such as patients with SCD. The focus groups revealed 5 themes that described best practices to promote engagement with a digital CBT program for mental health.

First, patients crave social connections and desire to feel a sense of belonging. Patients with SCD often express a fear of rejection when disclosing their SCD status and desire understanding and support from their peers. Symptoms of SCD impede engagement in social activities, employment, and school, and concerns regarding their peers’ perceptions may discourage individuals with SCD from establishing relationships or seeking support [32-34]. Both adolescents and adults recognize the importance of all forms of social support in their lives; however, social support from other individuals with SCD plays a unique role, because these connections provide the opportunity to empathize with each other’s experiences and share strategies and skills for coping with SCD.

The success and relevance of social support features in these apps have varied based on the target population [35-38], hence there is a need to evaluate the potential of interventions that increase social support to improve mental health among patients with SCD. Features that allow for increased social connection and encourage social identity among patients with SCD may yield additional mental health benefits that could lead to a further reduction in depression and other mental health disorders [39,40]. Social connections and support between peers living with SCD provide an opportunity to develop a social support network outside the app user’s family and health care team [35]. Many participants in this study recommended tools that allow connection with other app users with similar struggles and experiences. Incorporating components of social connections, such as digital support groups or peer connect features, may enhance the efficacy of digital CBT.

The presence of a health coach may be another way to foster social connection. Health coaching or real-time support has been shown to be a critical component of the effectiveness of digital CBT [30,31]. However, there is no evidence to detail how digital health coaching can be tailored to SCD or other minority populations. The adoption of techniques to ensure personalized treatment may be beneficial. These include using the patient’s name throughout, tailoring information specific to the individual user, including multiple modes of communication, and addressing patient concerns that are not directly related to disease pathology.

The literature varies, but several characteristics contribute to the effectiveness of peer coaches. Overall, it seems that the background, attitude, and empathy of the coach are the most important for building a relationship with the participant. Ideally, coaches should share a similar background with the participant, either sharing the same chronic illness or having a similar chronic illness that shares management skills, or the coaches should come from the same community as the participant [41]. Community health workers, who are also a source of social support and may encourage behavior change or provide coaching that supports behavior change, are the most effective when they share a common background with the community they serve [41]. In addition, prior research on other chronic disease populations suggests that successful peer coaches may not need to have high disease-related self-efficacy; in fact, peer coaches who have struggled may be less intimidating for participants and may be more successful in building rapport with their mentee [42].

Our participants also expressed mixed preferences regarding health coach qualifications, with some wanting coaching from a person with SCD, while others said that a professional would be preferred. The varied needs and expectations of patients with SCD may affect what kind of health coach is the most suitable. In particular, patients who lack peer social support may benefit greatly from a health coach who has experience in managing SCD whom they can confide in. Likewise, those who already have strong social networks may prefer professionals. Preferences for a peer versus professional coach could be tied to what concerns are most salient for the individual participant, for example an eHealth intervention that used advanced practice registered nurses (APRNs) found that patients were comfortable texting the APRN regarding physical symptoms but were less comfortable discussing personal problems, even when invited to share such experiences [43].

Participants in this study did not want the digital CBT intervention to focus exclusively on SCD as the sole source of depression, anxiety, or stress, and the ability to talk to a peer coach regarding personal problems unrelated to SCD may be crucial for some participants. However, it is worth noting that these preferences are not universal. Jacob et al [43] reported that some patients with SCD expressed appreciation knowing that the APRNs cared and were available to talk even though they may not have chosen to discuss personal problems, and some enjoyed having conversations with the APRNs regarding their personal life in addition to communicating regarding their SCD. Although patient preferences regarding coach qualifications may vary, this finding reinforces the importance of the coach’s interpersonal skills, whether they are a peer with SCD or a professional.

Given the variability in participants’ preferences for coaches, the consideration of each participant’s preferences during coach assignment could be beneficial in increasing engagement. Providing access to the background and expertise of health coaches may foster connections between users and coaches. For example, a successful study of peer coaches for patients with diabetes that saw improvements in hemoglobin A_1C_ used a booklet that included a photo and brief description of each coach to elicit participants’ preferences [42]. A similar guidebook could be used in the future to pair digital CBT SCD participants with their coaches. Our participants also expressed the importance of having one health coach as the main point of contact for digital CBT during the course of the intervention. This approach may also be crucial to maintaining engagement, as consistency gives both coach and participant time to build a trusting relationship, and gives the coach the opportunity to learn more about the individual participant and provide the personalized support that the participant desires. Attempting to match participants and coaches as much as possible based on availability and preference may help prevent attrition and increase participant engagement in the digital CBT intervention.

Journaling and symptoms tracking were features that patients felt were important for inclusion in a mental health app. Several SCD studies have implemented e-diaries that provide an ecological momentary assessment that quantitatively tracks real-time disease-related symptoms [22,44]. Several participants in this study indicated that quantitative tracking of symptoms, such as pain, activity, mood, and sleep, is beneficial and an app feature that they thought is important for understanding their health. However, participants also emphasized the importance of narrative journaling to fully understand the factors associated with their health and wellness. To the best of our knowledge, no study has implemented a narrative journaling feature in mobile mental health apps for SCD. Journal features may provide users with an alternative method for reflection and increase self-awareness while providing additional mental health benefits [45,46]. Expressive writing is a well-known therapeutic intervention with both physical and psychological health benefits [47]. Thus, including a journaling feature in mental health apps, implemented as open-ended text or voice entry, may not only help patients identify factors associated with their symptoms but may also provide a therapeutic effect via emotional disclosure. The developers of behavioral mental health apps for this population should consider not only having an e-diary feature that allows for quantitative tracking of symptoms but also a narrative journal feature that allows more open-ended expression and documentation of an individual’s day-to-day experiences.

### Limitations

The sample obtained for this study was relatively small, with one focus group having only 3 of the 10 scheduled participants in attendance. Thus, some data may not encompass the unique preferences and needs of this population. In addition, this study intentionally focused on individuals with SCD, with a sample age range primarily limited to individuals aged <30 years (only one participant was aged >30 years). Older individuals may have different perspectives and preferences than younger individuals. Therefore, although much of the presented information may apply to other chronic disease populations and age groups, careful consideration should be given when translating these findings to other populations.

### Digital CBT Recommendations and Conclusions

On the basis of the findings of this study, we have broad recommendations for designing digital CBT programs for adolescents and young adults with SCD.

#### Social Connection

Individuals with SCD look for ways to seek support from peers and find communities. Consider implementing a forum, support groups, scoreboards, or other features that allow users to connect, communicate, and see the activities of other participants within the digital CBT app.

#### Personalization

Users feel more motivated to use digital CBT programs when the apps feel more personalized. This includes automation that allows for personalization, for example, having a chatbot calling the user by name and the suggestion of topics that are relevant to the user based on prior activity. Features that the user can control and make their own, such as choices for the welcome screen or theme of the app, colors, creating an avatar, etc, also provide an opportunity for customization.

#### Health Coach

Digital CBT health coaches need to have a background knowledge regarding SCD (ie, some basic training and knowledge of the condition, exposure to community) as well as adequate training in mental health and behavioral treatments. A person with lived experience as a patient with SCD, as well as mental health training, would be ideal, but when not available, having a background knowledge regarding SCD may be sufficient. Personalized human support along with digital education is necessary.

#### Symptom Monitoring

Digital CBT apps should have features such as journals, logbooks, or other spaces with the capacity to monitor symptoms and daily thoughts. This allows the user to identify connections between daily events and mental and physical symptoms.

#### Content

For young adults and adolescents, it is important to have quick, engaging, and multimedia-rich content. Content should be “bite sized,” with enough new content and variety to satisfy diverse learning styles and information preferences among users. A one-size-fits-all approach has not been successful. Finally, the length of lessons should consider the cognitive load and competing demands on attention.

Optimizing digital CBT for individuals with SCD is vital for successful implementation of this intervention modality. Patients who perceive digital CBT as irrelevant, unrelatable, or culturally insensitive are less likely to initiate and sustain engagement in this treatment. The results of this study provide important insights into ways to design and implement digital CBT to advance the engagement in and effectiveness of digital CBT among individuals living with SCD. Personalizing the content delivered and coaching as much as possible, creating a desirable space for social connection between patients, and creating and delivering content that is relevant to patients with SCD while also acknowledging other contributors to mental health are all characteristics that will increase the successful implementation of digital CBT in routine SCD care. Researchers and developers should consider these findings as potential strategies for optimizing digital mental health support for this and other vulnerable populations.

## Abbreviations

APRN: advanced practice registered nurse
CBT: cognitive behavioral therapy
SCD: sickle cell disease

## Data Availability

Data supporting the findings of this study are available upon reasonable request from the corresponding author, CJ.
